# CPU Energy Meter: A Tool for Energy-Aware Algorithms Engineering

**DOI:** 10.1007/978-3-030-45237-7_8

**Published:** 2020-03-13

**Authors:** Dirk Beyer, Philipp Wendler

**Affiliations:** 8 Johannes Kepler University, Linz, Austria; 9 University of Birmingham, Birmingham, UK; LMU Munich, Munich, Germany

**Keywords:** Energy Measurement, RAPL, Benchmarking, BenchExec

## Abstract

Verification algorithms are among the most resource-intensive computation tasks. Saving energy is important for our living environment and to save cost in data centers. Yet, researchers compare the efficiency of algorithms still in terms of consumption of CPU time (or even wall time). Perhaps one reason for this is that measuring energy consumption of computational processes is not as convenient as measuring the consumed time and there is no sufficient tool support. To close this gap, we contribute CPU Energy Meter, a small tool that takes care of reading the energy values that Intel CPUs track inside the chip. In order to make energy measurements as easy as possible, we integrated CPU Energy Meter into BenchExec, a benchmarking tool that is already used by many researchers and competitions in the domain of formal methods. As evidence for usefulness, we explored the energy consumption of some state-of-the-art verifiers and report some interesting insights, for example, that energy consumption is not necessarily correlated with CPU time.

## 1 Introduction

There is a strong demand to save electrical energy, of which nowadays a large portion is used by computational processes. Most importantly, we need to protect the environment that we live in, but we also need to consider that energy usage is one of the most important cost factors in data centers: after computing devices are purchased and installed, the operational cost is dominated by the cost of consumed electrical energy. And since most of the used electrical energy is turned into heat energy, there is follow-up cost for the cooling system, which sets the limits of used energy for each rack in a data center [16].

In order to control energy consumption, we first need to measure it. Work in the area of green software engineering identified a lack of data and insufficient tool support [12]. Energy consumption of an algorithm is often reduced to CPU time, which seems to be a natural choice at a first look, but after more accurate measurement we know that this reduction leads to wrong conclusions.

Why is energy usage of verification algorithms not measured but only CPU time? Most likely it is technically too difficult for researchers to measure energy consumption, because it would require external hardware that is not common or because internal energy measurements are not well-known and complex to use.

In order to provide a solution to this problem, we contribute an open-source lightweight tool that enables convenient energy measurement for a large range of modern CPUs. The tool CPU Energy Meter makes it easy and convenient to access energy measurements done by the CPU for various of its parts. Furthermore, we integrate energy measurement in the benchmarking framework BenchExec, which is widely used by researchers and competitions (e.g., [2]).

Using CPU Energy Meter does not require any extra hardware, but accesses the existing feature for energy measurement called RAPL that Intel CPUs provide. This convenience comes with a limitation: We can only access measurement values for those parts of the computing board that the CPU measures, but no external equipment, such as hard drives and the power supply itself.

**Related Work.** Energy measurements should be used for algorithm engineering [1], and there is a strong need for tool support, such as PowerPack [8]. RAPL is being studied as a measurement method for energy consumption [6, 9, 10, 13, 17], and energy measurements that are based on RAPL are being developed for specific scenarios [11, 15, 18, 19] and used to evaluate algorithms [7]. CPU Energy Meter makes energy measurement conveniently accessible to verification researchers. The most closely related project is the Performance API (PAPI) analysis library, which also supports RAPL [19], but this is a large library with a much larger scope than just energy measurements. In contrast, our tool is a ready-to-use solution for energy measurements that is easy to install and use.

## 2 Intel Running Average Power Limit (RAPL)

The Intel Running Average Power Limit (RAPL) [14] is a feature of Intel CPUs that allows to measure and limit the energy consumption of CPUs. It is available since the $2^{\text{nd}}$ generation of the Intel Core architecture (code name "Sandy Bridge"), i.e., on Intel Core i3/i5/i7 2000 and newer, as well as Intel Xeon E3/E5/E7 CPUs. This covers a wide range of common CPUs for notebooks, desktops, and servers.

One part of RAPL consists of access to a series of hardware counters in which the CPU accumulates the energy it has consumed. RAPL supports measuring the energy consumption of so-called "domains", and up to five domains are supported by current CPUs: package, PP0, PP1, DRAM, and PSYS. Which hardware units are included in which domain is not clearly specified by Intel, but in general we can use the following assumption: The package domain refers to the whole CPU, the PP0 domain refers to the processor cores, and the PP1 domain refers to other units such as an integrated graphics unit. The domains DRAM and PSYS may provide information on the energy consumption of the RAM and other hardware on the mainboard, but both need special support from the hardware platform and its values may not be comparable between different systems.

There is no official information by Intel on the precision of the measurements except that the counters are updated approximately every 1 ms. The resolution of the values varies between the CPUs, but is typically $\frac{1}{2^{16}}$ J or $\frac{1}{2^{14}}$ J, i.e., in the order of $10^{-5}$ J. For the first generation of CPUs with RAPL, the energy consumption was approximated by the CPU and imprecise, but for subsequent generations the precision had been improved [6, 7, 10].

## 3 CPU Energy Meter

Our tool CPU Energy Meter provides access to the energy-measurement features of Intel CPUs to users. It was developed based on the tool Intel Power Gadget for Linux[1]. Our tool is available as open source under the permissive 2-clause BSD license and hosted on GitHub[2]. Installation packages of CPU Energy Meter are available for Debian-based distributions (e.g., Ubuntu).

CPU Energy Meter measures the energy consumption of the CPU(s) of a system for a specific time interval as reported by the RAPL interface (cf. Sect. 2). In order to ensure the highest possible measurement precision with the lowest possible overhead, it reads the RAPL energy counters as rarely as possible instead of using continuous sampling, while at the same time reading the counters often enough to safely detect and account for counter overflows. Furthermore, our tool was developed to use a minimal amount of necessary dependencies and permissions in order to make its installation as easy as possible.

**Requirements.** CPU Energy Meter requires a system with one or more Intel CPUs that support the RAPL feature. It needs direct access to the CPUs, thus running in a virtual machine is not supported. Accessing the model-specific registers of CPUs with the energy measurements is done via the Linux kernel module `msr`[3], which needs to be loaded and provides device files named `/dev/cpu/*/msr`.

Typically, access to these device files is granted only to the user `root`. In order to not need to execute CPU Energy Meter as `root`, one can change the file permissions of the device files appropriately (e.g., by granting read permissions to a group `msr` and making CPU Energy Meter always execute as this group using the "setgid" permission). Furthermore, CPU Energy Meter needs the capability `CAP_SYS_RAWIO`[4], which can be granted using `setcap`[5]. The installation packages of CPU Energy Meter attempt to automatically configure the system such that every user can execute the tool without granting any other non-standard permissions to users. In any case (whether executed as `root` or not), CPU Energy Meter drops all unnecessary permissions as soon as possible using the library "libcap"[6] in order to reduce any risk related to the non-standard permissions.

**Usage.** CPU Energy Meter is intended primarily to be used by benchmarking frameworks, however, manual execution is also possible. When the tool is executed, it starts the measurements and prints the consumed energy for all supported domains and CPUs of the system as soon as it is killed via the interrupt signal or Ctrl+C. Intermediate measurements are printed when the signal `USR1` is received. To manually measure the energy consumption of the duration of a specific command, one can execute the following command line, for example:

```
cpu-energy-meter & some_command ; kill -INT %1
```

1 https://software.intel.com/en-us/articles/intel-power-gadget
2 https://github.com/sosy-lab/cpu-energy-meter
3 http://man7.org/linux/man-pages/man4/msr.4.html
4 http://man7.org/linux/man-pages/man7/capabilities.7.html
5 http://man7.org/linux/man-pages/man8/setcap.8.html
6 https://sites.google.com/site/fullycapable/

This will measure the energy consumption of all CPUs during the whole time that the specified command is running, regardless of whether this energy consumption is caused by the specified command or by other processes running in parallel (this is a limitation of the RAPL feature). Thus, measuring the energy consumption during a specific time period (e.g., 10 s) can be done by replacing `some_command` with `sleep 10`.

The output values are given with the unit Joule, and can be formatted either in a way that is optimized for being read by humans (cf. Fig. 1) or parsed by programs.

```
+-----------------------------+
| CPU Energy Meter   Socket 0 |
+-----------------------------+
Duration           9.990624 sec
Package           15.898926 Joule
Core               1.695740 Joule
Uncore             0.352661 Joule
DRAM              11.024841 Joule
PSYS             104.778931 Joule
```

Fig. 1: Example output of CPU Energy Meter on a single-CPU system of the SkyLake generation (with all five domains supported)

**Integration into BenchExec.** We have contributed an integration of CPU Energy Meter into the benchmarking framework BenchExec [4], because BenchExec is widely used in the formal-methods community (e.g., SV-COMP [2]). Starting with version 1.16, BenchExec automatically executes CPU Energy Meter if the latter is installed, and it reports the energy results in the same manner as the results of its internal time and memory measurements (BenchExec supports the creation of CSV tables and interactive HTML tables with plots for its benchmarking results). BenchExec will report the energy consumption only if all cores of one or more CPUs are used for each tool execution, because we cannot distinguish between the energy consumption of individual processes.

## 4 Applications

The 8$^{th}$ International Competition on Software Verification (SV-COMP'19) [3] measured energy consumption of verification tools using BenchExec and CPU Energy Meter and for the first time provided an alternative "green" ranking based on energy efficiency (CPU-energy usage divided by achieved score). This ranking was indeed considerably different from the main score-based ranking, with no overlap between the top three green verifiers and the top three verifiers in the category "C-Overall". Furthermore, the winner in the green ranking is two orders of magnitude more efficient than the last tool in the ranking (64 J per score point vs. 4 200 J per score point). This shows an enormous potential of efficiency improvements and energy savings if verification researchers get access to easy measurements of energy usage.

In the following, we analyze in more detail some energy measurements of SV-COMP'19, which provides all raw results online [7]. We pick the results for the submissions Cbmc [8] and CPA-Seq [9] across all categories. CPA-Seq is the winner of the category "C-Overall", written in Java, and employs several different algorithms, some of which are partially parallelized. The garbage collector that

[7] https://sv-comp.sosy-lab.org/2019/results/results-verified/All-Raw.zip
[8] http://www.cprover.org/cbmc/ [9] https://cpachecker.sosy-lab.org/

Table 1: Selection of Energy Measurements from SV-COMP'19

| | CBMC | | | CPA-SEQ | | |
|---|---|---|---|---|---|---|
| RAPL domain | Package | PP0 (Core) | DRAM | Package | PP0 (Core) | DRAM |
| Average power used per task with regard to wall time (energy divided by wall time): | | | | | | |
| Min (W) | 1.9 | 1.2 | 0.63 | 4.4 | 3.4 | 1.6 |
| Max (W) | 25 | 24 | 5.5 | 36 | 35 | 7.2 |
| Avg (W) | 9.7 | 8.8 | 2.4 | 20 | 19 | 2.8 |
| Std. Dev. (W) | 3.2 | 3.2 | 0.71 | 6.2 | 6.2 | 0.48 |
| Average power used per task with regard to CPU time (energy divided by CPU time): | | | | | | |
| Min (W) | 1.8 | 1.1 | 0.58 | 4.2 | 3.2 | 0.70 |
| Max (W) | 23 | 22 | 5.5 | 17 | 16 | 6.8 |
| Avg (W) | 9.6 | 8.7 | 2.4 | 9.6 | 9.0 | 1.5 |
| Std. Dev. (W) | 3.1 | 3.1 | 0.74 | 1.8 | 1.7 | 0.60 |

is used by the JVM adds some more parallelism. CBMC is written in C++ and uses bounded model checking in a strictly sequential implementation. Thus, we expect that the energy consumption of these tools has different characteristics. SV-COMP'19 executed both tools for 10 522 tasks (CPU-time limit 900 s per task, Intel Xeon E3-1230 v5 CPU, quad-core with hyper-threading, 3.4 GHz, all 8 processing units of the CPU and 15 GB of memory were available to each tool execution, Ubuntu 18.04 64-bit with Linux kernel 4.15 was the operating system).

We now compare the energy consumption of the RAPL domain "Package" with the CPU time for CBMC in Fig. 2 and for CPA-SEQ in Fig. 3.[10] In the plot, all results that lie on the same line through the origin belong to tool executions for which the energy consumption per second of CPU time (in $\frac{\mathrm{J}}{\mathrm{s}} = \mathrm{W}$) was the same (this would be the average power of the CPU if measuring wall time instead of CPU time). We provide additional statistics in Table 1 and two graphs that compare the CPU time and the energy consumption of the two tools in Fig. 4.

**Insight:** *Also for verification tools, high values for CPU time do not imply high values for energy.* Figure 2 has a large vertical area of data points where the CPU time is close to the time limit. For those verification runs, the energy is in the range of 2.0 kJ to 15 kJ. This shows that for a specific CPU time, the energy consumption (and average power, cf. Table 1) for different verification tasks can vary by a factor of 7.

**Insight:** *Comparing different verification tools regarding CPU time can lead to different conclusions than energy-based comparisons.* The graph on the left of Fig. 4 compares CBMC and CPA-SEQ regarding CPU time, the graph on the right compares them regarding energy consumption. The difference between the shapes of these two graphs shows that looking at the energy consumption when comparing tools is an interesting addition to comparing only CPU time, and that

[10] For CPA-SEQ, the CPU time is sometimes higher than 900 s because SV-COMP lets tools optionally run for more than the time limit in order to print additional statistics (but any result after the time limit is of course discarded).

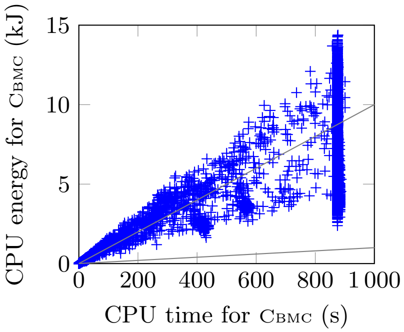

Fig. 2: Comparison of CPU time vs. energy consumption for Cbmc

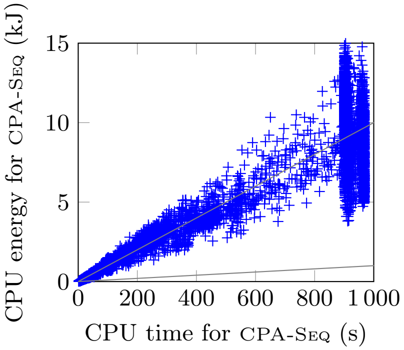

Fig. 3: Comparison of CPU time vs. energy consumption for CPA-Seq

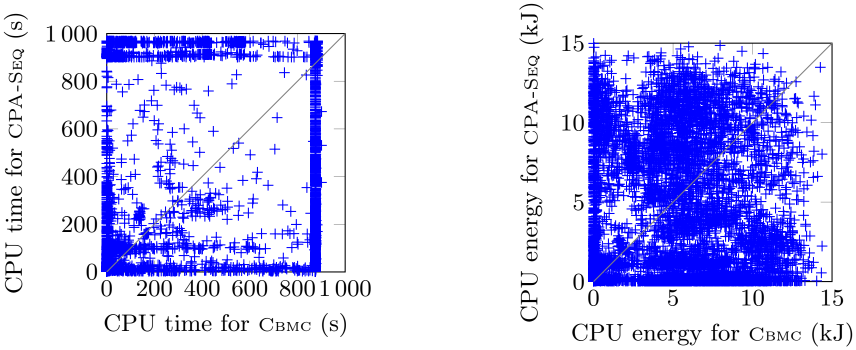

Fig. 4: Comparison of Cbmc and CPA-Seq with regard to CPU time and energy the similar statistics on power usage with regard to CPU time (cf. lower part of Table 1 and Figs. 2 and 3) can be misleading: if the power-usage characteristics of both tools were the same, the two graphs in Fig. 4 would look similar.

## 5 Conclusion

Verification algorithms consume large amounts of energy and thus, it is prohibitive to ignore the energy characteristics of algorithms when comparing their quality. Although this matter is understood, the verification community does not measure energy. We believe that this is because measurement of energy is complex and requires a lot of additional effort. The lightweight tool CPU Energy Meter fills this gap: It supports reading Intel-RAPL-based energy measurements in a convenient way and —via integration into BenchExec— using a tool environment that many verification researchers use anyway already.

An analysis of a large data set from a verification competition invalidates a wide-spread assumption: the data quickly reveal that energy consumption can deviate significantly from the consumed CPU time. Thus, it is not sufficient to measure CPU time.

**Data Availability Statement.** A replication package for this article including CPU Energy Meter and BenchExec is available at Zenodo [5]. Current versions of CPU Energy Meter are available at https://github.com/sosy-lab/cpu-energy-meter and https://doi.org/10.5281/zenodo.1300309. The dataset from SV-COMP'19 [3] that was analyzed in Sect. 4 is available online at https://sv-comp.sosy-lab.org/2019/results/results-verified/All-Raw.zip.
